# Enhanced CT-Based Radiomics to Predict Micropapillary Pattern Within Lung Invasive Adenocarcinoma

**DOI:** 10.3389/fonc.2021.704994

**Published:** 2021-08-27

**Authors:** Yunyu Xu, Wenbin Ji, Liqiao Hou, Shuangxiang Lin, Yangyang Shi, Chao Zhou, Yinnan Meng, Wei Wang, Xiaofeng Chen, Meihao Wang, Haihua Yang

**Affiliations:** ^1^Key Laboratory of Radiation Oncology of Taizhou, Radiation Oncology Institute of Enze Medical Health Academy, Department of Radiation Oncology, Taizhou Hospital Affiliated to Wenzhou Medical University, Taizhou, China; ^2^Department of Radiology, The First Affiliated Hospital of Wenzhou Medical University, Wenzhou, China; ^3^Department of Radiology, Taizhou Hospital of Zhejiang Province Affiliated to Wenzhou Medical University, Taizhou, China; ^4^Department of Radiation Oncology, University of Arizona, Tucson, AZ, United States; ^5^Department of Radiation Oncology, Indiana University School of Medicine, Indianapolis, IU, United States

**Keywords:** lung adenocarcinoma, micropapillary pattern, radiomics, early diagnosis of cancer, computer tomography

## Abstract

**Objective:**

We aimed to investigate whether enhanced CT-based radiomics can predict micropapillary pattern (MPP) of lung invasive adenocarcinoma (IAC) in the pre-op phase and to develop an individual diagnostic predictive model for MPP in IAC.

**Methods:**

170 patients who underwent complete resection for pathologically confirmed lung IAC were included in our study. Of these 121 were used as a training cohort and the other 49 as a test cohort. Clinical features and enhanced CT images were collected and assessed. Quantitative CT analysis was performed based on feature types including first order, shape, gray-level co-occurrence matrix-based, gray-level size zone matrix-based, gray-level run length matrix-based, gray-level dependence matrix-based, neighboring gray tone difference matrix-based features and transform types including Log, wavelet and local binary pattern. Receiver operating characteristic (ROC) and area under the curve (AUC) were used to value the ability to identify the lung IAC with MPP using these characteristics.

**Results:**

Using quantitative CT analysis, one thousand three hundred and seventeen radiomics features were deciphered from R (https://www.r-project.org/). Then these radiomic features were decreased to 14 features after dimension reduction using the least absolute shrinkage and selection operator (LASSO) method in R. After correlation analysis, 5 key features were obtained and used as signatures for predicting MPP within IAC. The individualized prediction model which included age, smoking, family tumor history and radiomics signature had better identification (AUC=0.739) in comparison with the model consisting only of radiomics features (AUC=0.722). DeLong test showed that the difference in AUC between the two models was statistically significant (P<0.01). Compared with the simple radiomics model, the more comprehensive individual prediction model has better prediction performance.

**Conclusion:**

The use of radiomics approach is of great value in the diagnosis of tumors by non-invasive means. The individualized prediction model in the study, when incorporated with age, smoking and radiomics signature, had effective predictive performance of lung IAC with MPP lesions. The combination of imaging features and clinical features can provide additional diagnostic value to identify the micropapillary pattern in IAC and can affect clinical diagnosis and treatment.

## Introduction

Lung cancer is the most common cause of cancer-related death worldwide. Non-small cell lung cancer (NSCLC) accounts for approximately 85% of all cases of lung cancer, and with the most common histological subtype of NSCLC being adenocarcinoma (1). Currently, surgical resection is the main treatment for lung adenocarcinoma (2).

According to the 2015 World Health Organization Classification of Lung Cancer (3), based on prognosis, lung adenocarcinoma is classified into five histopathological subtypes. Micropapillary type is a high-risk subtype which has the potential for rapid metastasis and a poor prognosis (4, 5). Micropapillary pattern (MPP) refers to the free central cluster of cells lacking fibrous vessels (6). It has been reported in published literature that the micropapillary pattern of lung adenocarcinoma also has a negative effect on prognosis and survival after postoperative radiotherapy and chemotherapy (7, 8). According to Lee G et al., even a small proportion of MPP specifically <5% of the entire tumor volume has a significant negative prognostic impact on the overall survival (OS). Thus, the negative prognostic impact of MPP plays an important role in the clinical treatment plan and the early diagnosis of MPP in the lung IAC become very important (9).

Currently, the common preoperative diagnostic methods of lung cancer are histological puncture biopsy and CT imaging methods. However, histological biopsies are invasive and conventional CT imaging diagnostic methods are subjective and qualitative (10, 11). Moreover, most lung adenocarcinomas do not have high-grade components, and so radiographic features may not be obvious in identifying these cases (12–14).

Radiomics is a non-invasive and quantitative methods for describing the biological characteristics and heterogeneity of the tumor and we aimed to use this for the early diagnosis of pulmonary micropapillary lesions. Radiomics has become one of the research hotspots due to its high patient-specific and non-invasive comprehensive advantages. Possibly, radiomics has potential application for the precise treatment of NSCLC. Radiomics is expected to be used to judge biological characteristics of tumors such as benign or malignant pulmonary nodular, lymph node metastasis and tumor gene phenotype, to reflect tumor response to treatment, and to predict prognosis and radiation-induced lung injury and provide an objective basis for individualized treatment (11, 15, 16). This study is based on the features from CT imaging data over a given region of interest (ROI).

Previously, we investigated the diagnosis of micropapillary type of lung adenocarcinoma to some extent by radiomic methods (17) and the purpose of the current study is to further study the use of CT-based radiomics to preoperationally predict MPP in lung invasive adenocarcinoma.

## Materials and Methods

### Patients

**Figure 1:**
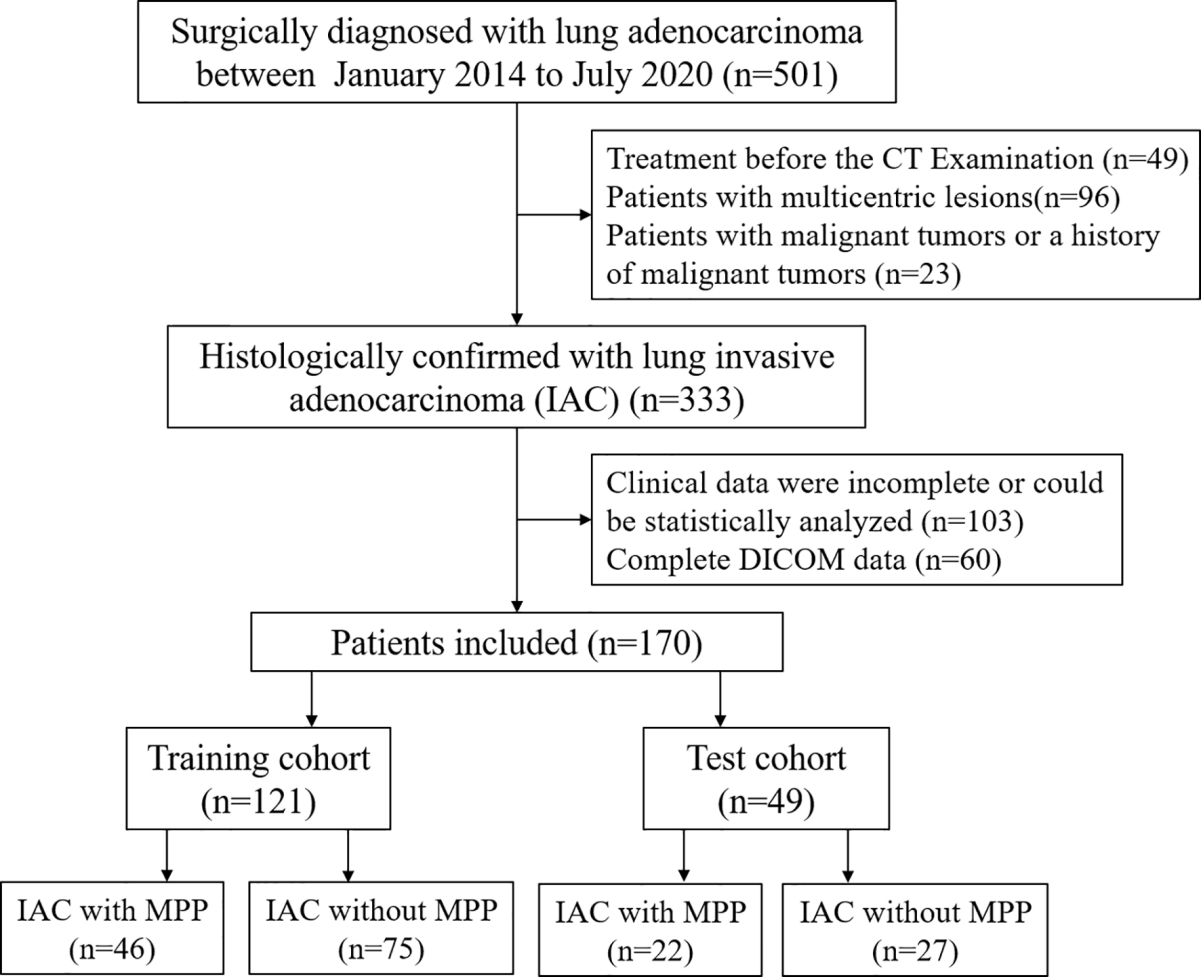
The flow chart of patient selection.

A total of 170 patients between July 2014 and July 2020 were included in our study. 121 cases of single lung adenocarcinoma diagnosed by pathology were included as the training cohort, including 46 cases with MPP and 75 cases without MPP, aged 45-87 (67.7 ± 8.8) years old. Inclusion criteria: (1) no treatment before enhanced CT examination; (2) complete surgical resection with pathological confirmation; (3) complete DICOM data. Exclusion criteria: (1) clinical data were incomplete or could not be statistically analyzed; (2) preoperative chemotherapy or radiotherapy; (3) other malignant tumor or a history of malignant tumor; (4) radiomics analysis of CT images was not possible. In addition, forty-nine cases of single lung adenocarcinoma were collected and used as an independent testing cohort, including 22 cases with MPP and 27 cases without MPP, aged 26-85 (63.2 ± 12.1) years old. Inclusion criteria and exclusion criteria were the same as the training cohort. The flow chart is shown in **Figure 1**. The study is approved by the ethics committee of the author’s medical institution and informed consent was obtained.

### Image Acquisition and Segmentation

Helical CT images were gained by a 16-row CT scanner (GE Ultra) after deep inhalations. The examination parameters were as follows: voltage, 120KV; tube current, 150mA; layer thick, 1.25mm; and matrix, 512×512mm. A power syringe was used to inject contrast agent (60-85ml iodioxitol or iodipamil) at a rate of 2.5-3.0ml/s, and rapid scanning was performed after a delay of 30 seconds to obtain arterial CT images for the study. Two radiologists read the images and sketched the ROI layer independently and blindly with each other using ITK-SNAP (www.itksnap.org/). Then radiomic features were extracted from those volumes for further analysis.

### Clinical Data Collection

Clinical data were collected *via* electronic medical records. Clinical features were assessed at the time of diagnosis. Gender, age, smoking status, drinking status, family tumor history, UICC stage, type of surgery, and treatment history were recorded. Clinic characteristics are shown in **Table 1**.

**Table 1:** Patient characteristics.

Variables	Training cohort (N=121)	Test cohort (N=49)
Age, mean ± SD (range)	67.7 ± 8.8 (45-87)	63.2 ± 12.1 (26-85)
Gender, n		
Female	52	33
Male	69	16
Smoker, n	34	10
Family tumor history, n	11	11
Histologic subtypes within a tumor, n		
One subtype		
Acinar pre*	38	17
Micropapillary pre	5	1
Solid	7	0
Papillary	2	0
Adherent	14	4
Two subtypes		
Acinar + micropapillary	20	13
Adherent + micropapillary	1	0
Solid + micropapillary	3	2
Papillary + micropapillary	2	1
Adherent + papillary	1	1
Acinar + papillary	6	2
Acinar + solid	1	1
Acinar + adherent	6	1
Three subtypes		
Acinar + papillary + micropapillary	8	0
Adherent + papillary + micropapillary	1	1
Acinar + solid + micropapillary	6	3
Acinar + adherent + micropapillary	0	1
Acinar + adherent + solid	0	1
TNM stage*, n		
IA	80	36
IB	11	4
IIA	1	0
IIB	12	5
IIIA	14	2
IIIB	3	0
IV	0	2

*“pre” means to take the ingredient as the main ingredient.

*TNM stage is based on the eighth edition lung cancer TNM staging of International Association for the Study of Lung Cancer (IASLC) (18).

### Extraction of Radiomics Characteristics

Artificial Intelligent Kit (A.K., version V3.2.0. R) was used for image processing and feature extraction. The original DICOM images and the ROI files were separately resampled by the Image Preprocessing module of A.K., and the resampling voxel size was set as 1.000 × 1.000 × 1.000. Then the two types of enhanced CT images were matched to extract radiomics features which included features types and transform types by A.K. platform. The former types included first order, shape, gray-level co-occurrence matrix (GLCM), gray-level size zone matrix (GLSZM), gray-level run length matrix (GLRLM), gray-level dependence matrix (GLDM) and neighboring gray tone difference matrix (NGTDM), and the latter types included log, wavelet and local binary pattern (LBP).

### Selection of Relevant Features and Establishment of Individualized Prediction Model

The least absolute shrinkage and selection operator (LASSO) was applied for dimensionality reduction screening to produce reproducible and stable features that would be used to screen for the predictive performance studies. And the “corrplot” package was used for correlation analysis (**Supplementary Material**). The cor-function of R software was used to further analyze the potential correlation between radiomics features and clinical features in the differentiation of MPP within lung adenocarcinoma. Finally, the most relevant features were selected and used to develop prediction models for MPP of lung IAC.

### Statistical Method

R software (version 4.0.0, http://www.Rproject.org) was used for statistical analysis. The “glmnet” and “pROC” packages were used for LASSO reduction and ROC curve plotting. The LASSO method was used for features dimensionality reduction and the selection of the discriminative features. The cor-function was used to detect whether the differences of variables or indicators among different classifications and different sets were statistically significant. Intra-class correlation coefficient (ICC) values were compared to estimate the reproducibility and stability of tumor segmentation and feature extraction (ICC > 0.75 indicating good consistency). The receiver operating characteristic (ROC) curve and area under the curve (AUC) are used to evaluate the predictive effectiveness of models, and DeLong test was used to compare whether the efficiency differences between the models were statistically significant. Calibration curves were used to evaluate the correction effect of the nomograms.

## Results

### Extraction and Selection of Radiomics Features and the Establishment of Radiomics Signature

**Figure 2:**
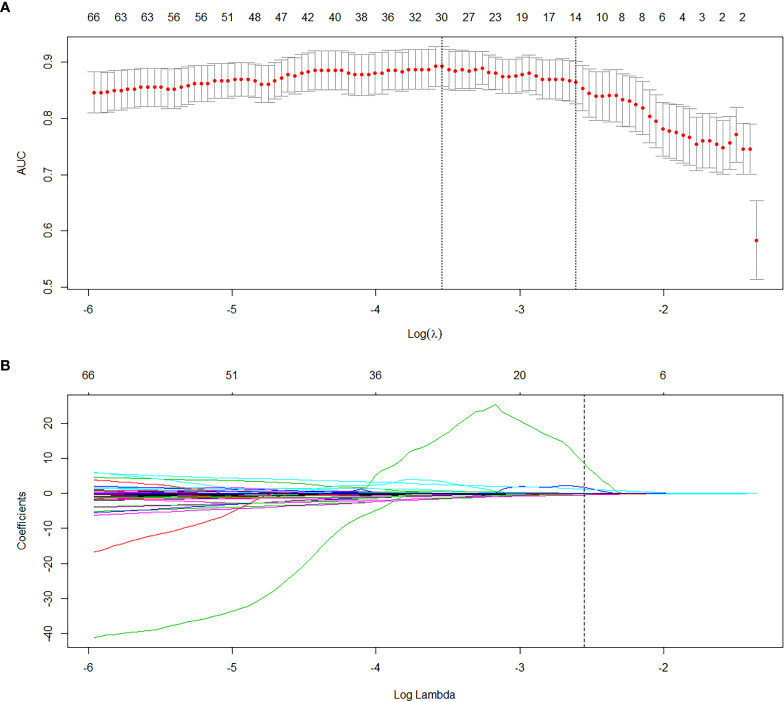
Dimension reduction to find 14 relevant radiomics features by LASSO. **(A)** The cross validation was performed by LASSO regression and the parameter λ was adjusted to find the best set of functions. The vertical dotted line on the left indicates that the logarithm (λ) corresponds to the optimal λ. The selection criterion is the minimum deviation value. **(B)** The texture parameter coefficients varied with λ. The vertical line represents the 14 features selected when the LASSO cross validation coefficients is non-zero. (λ=0.074).

**Figure 3:**
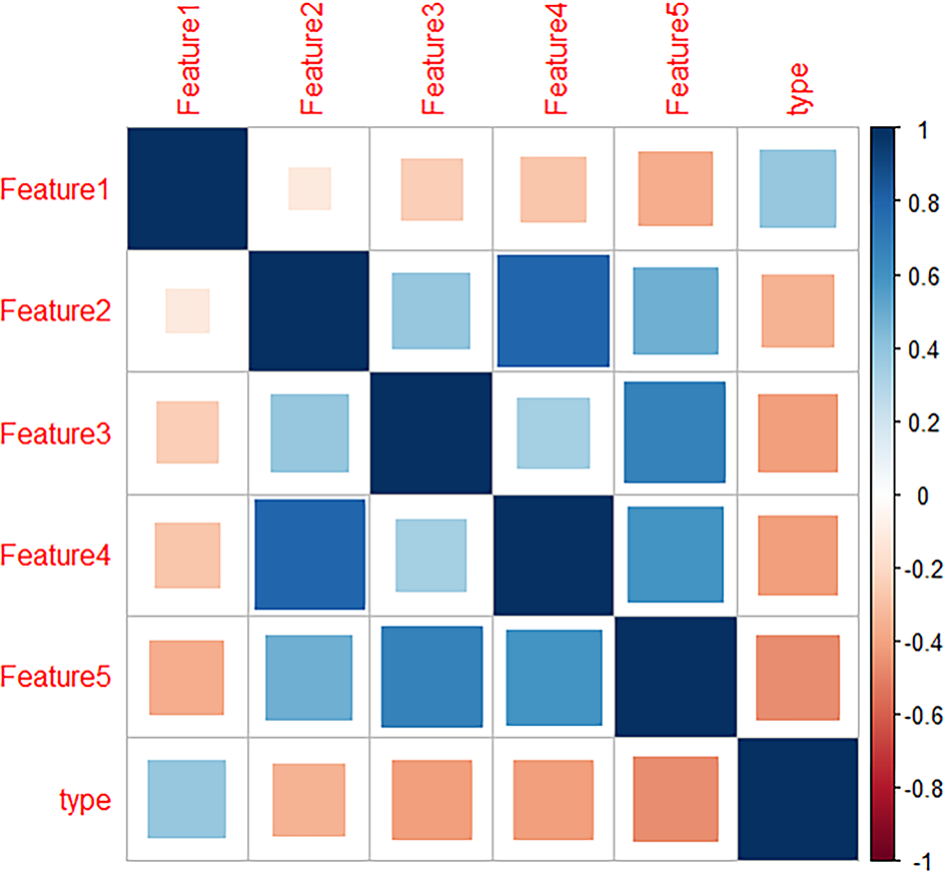
Heatmap of radiomics features which showed the correlation between the 5 radiomics features and lung invasive adenocarcinoma (IAC) with micropapillary pattern (MPP).

Data preprocessing was performed by R software. After deleting meaningless and empty data, 1317 radiomics features remained. Then, LASSO was used for dimension reduction of these features and the resulting lambda.1se=0.074 is obtained (**Figures 2A, B**). According to the results, 14 relevant radiomic features were selected. Then correlation analysis was conducted for the 14 features by the cor-function of R software and 5 key features were selected (**Figure 3**). They are referred to in this article as “Features 1-5”, as shown in **Table 2**.

**Table 2:** Five characteristic predictive parameters in the radiomics signature.

Code name	Full name	MEAN (SD)	W value	P value
Feature 1	wavelet.LLL_glszm_LowGrayLevelZoneEmphasis	0.39 (0.19)	1855.00	<0.01
Feature 2	wavelet.LHL_glrlm_LongRunLowGrayLevelEmphasis	4.21 (1.22)	5040.00	<0.01
Feature 3	wavelet.LLH_firstorder_RobustMeanAbsoluteDeviation	9.68 (4.52)	5156.00	<0.01
Feature 4	wavelet.LHL_glrlm_LongRunEmphasis	6.14 (1.39)	5198.00	<0.01
Feature 5	original_firstorder_InterquartileRange	18.9 (9.23)	5372.00	<0.01

### Predictive Efficacy of Radiomics Signature

**Figure 5:**
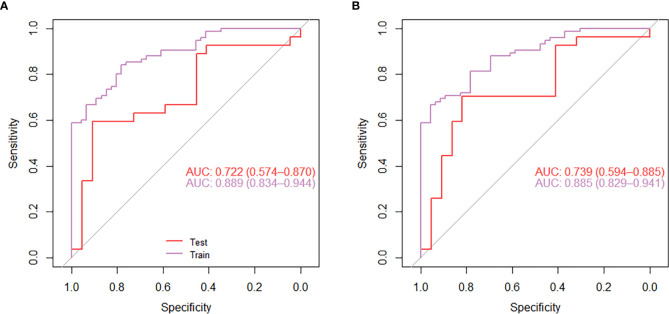
The receiver operating characteristic curve (ROC) analysis of the effect of the radiomics model nomogram **(A)** and the individual diagnostic prediction model nomogram **(B)** to predict micropapillary pattern within lung invasive adenocarcinoma.

The mean of each feature of the radiomics signature is shown in **Table 2**. Correlation test showed that the median difference of radiomics signatures between the lung IAC with MPP and lung IAC with non-MPP was statistically significant (P<0.05). As shown in **Figure 5A**, the efficacy of radiomics signature for differential diagnosis was good in the training cohort (AUC = 0.889, 95% CI: 0.843 – 0.944) and in the test cohort (AUC = 0.722, 95% CI: 0.574 – 0.870).

### Establishment of Individualized Diagnostic Prediction Model

**Figure 4:**
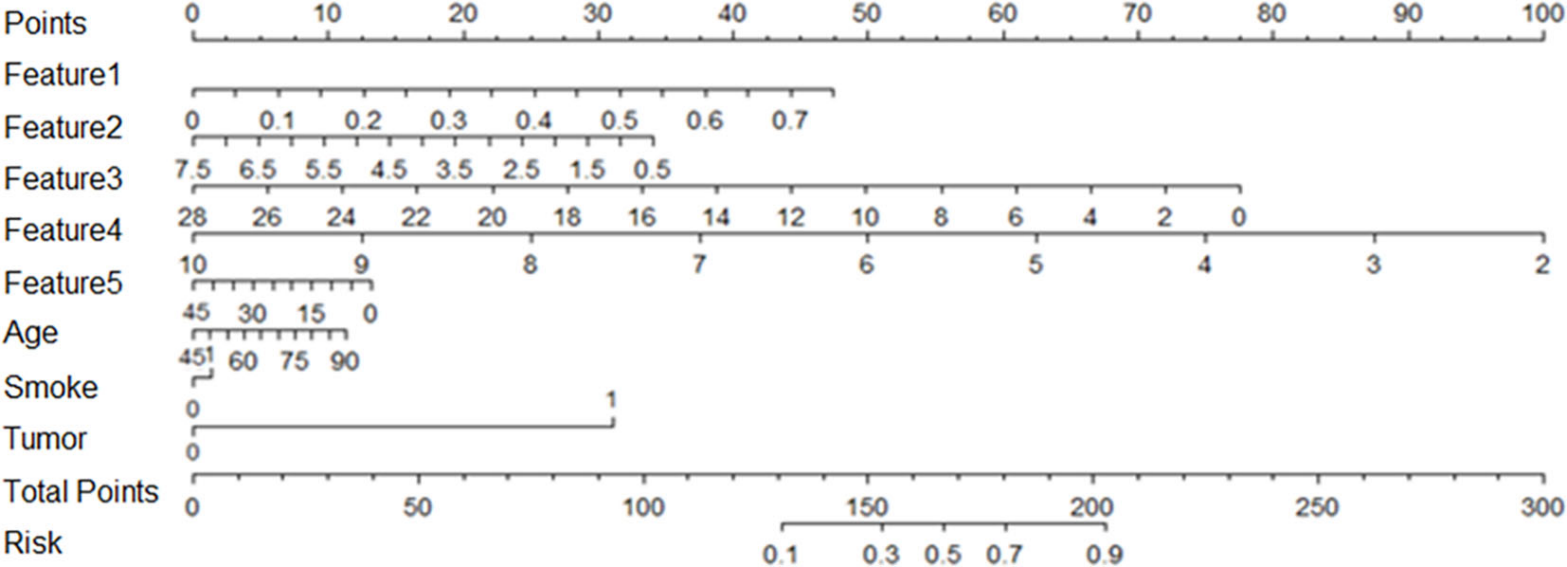
Based on the training cohort data, the nomogram of radiomics was established, and the statistical analysis showed that the nomogram consisted of radiomics signature, age, smoking and family tumor history, which could predict the risk of micropapillary pattern within lung invasive adenocarcinoma.

Correlation analysis was conducted for clinical features including age, gender, smoking status, drinking status and family tumor history, which was the same as the radiomic features selection above. There was no statistically significant difference in gender and drinking between the lung IAC with MPP and lung IAC with non-MPP in the training cohort and the test cohort. These clinically significant features were combined with the previously selected radiomic features to establish a personalized diagnostic prediction model for lung IAC with MPP, which is shown by a nomogram **Figure 4**.

### Comparison of Predictive Performance Between Radiomics Model and Individualized Diagnostic Model

For the individualized diagnostic model, the AUC in the training cohort was 0.885 (95% CI: 0.829 – 0.941), and in the test cohort, the AUC was 0.739 (95% CI: 0.594 – 0.885) (**Figure 5B**). In the test cohort, the diagnostic prediction effect of the individualized diagnostic model was relatively better than that of the radiomics model alone.

## Discussion

Recently, there has been some reports that lung IACs with MPP are associated with poor prognosis, such as the possible propensity for recurrence and metastasis (11, 19). MPP is considered to be associated with a high rate of lymphatic invasion, visceral pleural invasion and lymph node metastasis as well (20–23). Previous studies have shown that patients with neoplasms with an MPP component of 5% or more have a higher recurrence rate after limited resection than similar patients with lobectomies (24), suggesting that MPP may influence the choice of surgical approach. Therefore, preoperatively diagnosing lung IAC with MPP is very important for the clinic.

CT is the most widely applied method to diagnose lung cancer, but it is subjective for doctors to judge the nature of lesions and the sensitivity and specificity of detecting lymph node metastasis is also low (25, 26). Radiomics provides an more objective method for diagnosis. Recent developments in radiomics have made it possible to extract high-throughput imaging features, making it easier to obtain more accurate information about individual patients and their treatment options. Many studies have developed models of different clinical features for prediction based on radiomics, including prognosis and the conditions of lymph node metastasis. In addition, some researchers believe that radiomics analysis is valuable in predicting the clinical characteristics and molecular background of brain tumors (27–32). Our study also shows that radiomic features can be applied to determinate lung adenocarcinoma with MPP from other lung IAC and it is helpful not only for follow-up therapy but also suggests that radiomic analyses could be used to guide the selection of therapy for specific tumors (27). Radiomics may have a bigger role in the future.

We have studied the diagnosis of micropapillary type of lung IAC by means of radiomics before (17), and this study is a supplement and extension on the basis of the previous research. In this study, we investigated radiomics features to differentiate lung IAC with MPP from lung IAC with non-MPP based on enhanced CT images. In our previous study, only radiomics signature based on CT images were obtained, and this study added a comprehensive model combining clinical features and a comparison between the two models. The results showed that the radiomics features of CT images may be used to identify micropapillary pattern of lung invasive adenocarcinoma, and that the predictive efficiency of the personalized diagnostic model combined with clinical features was better than that of the omics label alone.

The clinical sample selection of our study is closely linked to postoperative pathology, which shows the reliability of the research results. CT images were used validate the radiomics nomogram. Reliable imaging characteristics are necessary for radiomics analysis (28). Previous studies have selected certain 3D imaging features and put them into application (33, 34). They showed the visual representation of tumors (27, 28). To construct the radiomic features, the correlation of prediction results was detected by LASSO, and the large numbers of radiomic features were reduced to 14 potential predictors. This method is not only better than the method of selecting predictors based on the univariate association strength of predictors and results, but also can combine the selected feature panels for performing predictive function (30).

There are some limitations in our study. First, this was a single-center study with a lack of external validation. Secondly, the sample size needs to be expanded for more reliable results. Third, texture features were extracted from manually segmented data, which made it difficult to exclude small vessels and bronchus inside the lesions, and may affect the accuracy of some features, and the development of a more reliable and repeatable automated segmentation method seems necessary. Moreover, the current study did not include genomics studies. It has been reported that radiogenomics can offer a practical way to leverage limited and incomplete data to generate knowledge that may lead to improved decision making, and as a result, improve patient outcomes (35). Other studies have shown that EMT-related molecules, EGFR, KRAS and Ki67 have some relationship with MPP (36–38). In the future, we will combine the genetic characteristics for further research and exploration.

In conclusion, radiomics has an important role to play in the diagnosis of tumors. The individualized predictive model, which consists of clinical features and radiomic signatures, expressed in the form of a histogram, can provide a non-invasive, rapid, inexpensive, and reproducible method for the individualized preoperative prediction of micropapillary pattern within lung invasive adenocarcinoma to determine the presence of micropapillary components and facilitate clinical management.

## Data Availability Statement

The original contributions presented in the study are included in the article/**Supplementary Material**. Further inquiries can be directed to the corresponding authors.

## Ethics Statement

The studies involving human participants were reviewed and approved by the Ethics Committee of Taizhou Enze Medical Center (Group) Taizhou Enze Medical Center (Group). The patients/participants provided their written informed consent to participate in this study.

## Author Contributions

YX, HY, and MW contributed to conception and design of the study. WJ, CZ, and WW organized the database. LH, SL, and YM performed the statistical analysis. YX wrote the first draft of the manuscript. YS and XC wrote sections of the manuscript. All authors contributed to the article and approved the submitted version.

## Funding

This work was supported in part by National Natural Science Foundation of China (CN) (NSFC 81874221).

## Conflict of Interest

The authors declare that the research was conducted in the absence of any commercial or financial relationships that could be construed as a potential conflict of interest.

## Publisher’s Note

All claims expressed in this article are solely those of the authors and do not necessarily represent those of their affiliated organizations, or those of the publisher, the editors and the reviewers. Any product that may be evaluated in this article, or claim that may be made by its manufacturer, is not guaranteed or endorsed by the publisher.
