# Supplementary material for: Enhanced CT-Based Radiomics to Predict Micropapillary Pattern Within Lung Invasive Adenocarcinoma
Source: Front Oncol. 2021 Aug 27;11:704994. doi: 10.3389/fonc.2021.704994 (PMC8429899; doi:10.3389/fonc.2021.704994)
Supplement: Supplementary file 1 [file DataSheet_1.pdf]

```
library(glmnet)
library(data.table)

LASSO=da[1:121,]
text_da=da[122:170,]
x=LASSO[,2:1317]
y=LASSO[1:121,1]
x=as.matrix(x)
set.seed(1)

fit <- glmnet(x, y, alpha=1,family = 'binomial',lower=-10, upper=10)
set.seed(1)
fit_cv <- cv.glmnet(x, y, alpha=1, family = 'binomial',
type.measure='auc')
plot(fit_cv)

get_coe <- function(the_fit,the_lamb){
  Coefficients <- coef(the_fit, s = the_lamb)
  Active.Index <- which(Coefficients != 0)
  Active.Coefficients <- Coefficients[Active.Index]
  re <-
data.frame(rownames(Coefficients)[Active.Index],Active.Coefficients)
  re <- data.table('var_names'=rownames(Coefficients)[Active.Index],
                   'coef'=Active.Coefficients)
  re$expcoef <- exp(re$coef)
  return(re[order(expcoef)])
}
get_coe(fit_cv,fit_cv$lambda.1se)
get_plot<- function(the_fit,the_fit_cv,the_lamb,toplot = seq(1,50,2)){
  Coefficients <- coef(the_fit, s = the_lamb)
  Active.Index <- which(Coefficients != 0)
  coeall <- coef(the_fit, s = the_fit_cv$lambda[toplot])
  coe <- coeall[Active.Index[-1],]
  ylims=c(-max(abs(coe)),max(abs(coe)))
  sp <- spline(log(the_fit_cv$lambda[toplot]),coe[1,],n=100)
  plot(sp,type='l',col =1,lty=1,
       ylim = ylims,ylab = 'Coefficient', xlab = 'log(lambda)')
  abline(h=0)
  for(i in c(2:nrow(coe))){
    lines(spline(log(the_fit_cv$lambda[toplot]),coe[i,],n=1000),
          col =i,lty=i)
  }
  legend("bottomright",legend=rownames(coe),col=c(1:nrow(coe)),
         lty=c(1:nrow(coe)),
```

```
        cex=1.2)
}
get_plot(fit,fit_cv,exp(log(fit_cv$lambda.1se)-1))



plot(fit)
plot(fit, xvar = "lambda", label = F)
abline(v=log(0.07357213),lty=2)

#####################################################

library(corrplot)
a=get_coe(fit_cv,fit_cv$lambda.1se)$var_names

select_name=c("type","lbp.3D.m2_firstorder_90Percentile","wavelet.LHL_
glrlm_LongRunEmphasis"
               , "wavelet.LLH_glcm_Contrast"
               ,"wavelet.LLH_firstorder_Skewness"
              ,"lbp.3D.m2_firstorder_RootMeanSquared"
               ,"original_firstorder_InterquartileRange"
               ,"wavelet.LLH_firstorder_RobustMeanAbsoluteDeviation"
               ,"wavelet.LHL_glrlm_LongRunLowGrayLevelEmphasis"
               ,"log.sigma.2.0.mm.3D_glszm_GrayLevelNonUniformity"
               ,"diagnostics_Image.original_Size"
               , "wavelet.LLL_firstorder_Kurtosis"
               , "wavelet.LLL_glszm_LowGrayLevelZoneEmphasis"
               ,"wavelet.LHL_firstorder_Entropy","wavelet.LHL_gldm_Gra
yLevelVariance")

select_da=da[select_name]

factor_Corr <- cor(select_da)
corrplot(factor_Corr,method="number")
corrplot.mixed(factor_Corr)

corrplot(factor_Corr, method = "square")


cor_name=c("wavelet.LLL_glszm_LowGrayLevelZoneEmphasis",
           "wavelet.LHL_glrlm_LongRunLowGrayLevelEmphasis",
           "wavelet.LLH_firstorder_RobustMeanAbsoluteDeviation",
           "wavelet.LHL_glrlm_LongRunEmphasis",
           "original_firstorder_InterquartileRange",
```

```
          "type","Age","Smoke","Alcohol")


cor_da=da[cor_name]

factor_Corr1 <- cor(cor_da2)
corrplot(factor_Corr1,method="number")
corrplot.mixed(factor_Corr1)
corrplot(factor_Corr1, method = "square")
setwd("D:\\TZhosiptal\\2021\\2021.2.1xyy\\2.22")
write.csv(cor_da, file = "cor_da.csv")
########################################


library(rms)

train_da=cor_da[1:121,]
text_da=cor_da[122:170,]

dd=datadist(train_da)

options(datadist="dd")


f1=lrm(type ~ wavelet.LLL_glszm_LowGrayLevelZoneEmphasis+
          wavelet.LHL_glrlm_LongRunLowGrayLevelEmphasis+
          wavelet.LLH_firstorder_RobustMeanAbsoluteDeviation+
          wavelet.LHL_glrlm_LongRunEmphasis+
          original_firstorder_InterquartileRange,data = train_da)


nom <- nomogram(f1, fun=plogis, lp=F,
funlabel="Risk",fun.at=c(0.1,0.5,0.9))
plot(nom)
```
